# Attentional control data collection: A resource for efficient data reuse

**DOI:** 10.3758/s13428-025-02717-z

**Published:** 2025-06-24

**Authors:** Julia M. Haaf, Madlen Hoffstadt, Sven Lesche

**Affiliations:** 1 University of Potsdam, Karl-Liebknecht-Str. 24/25, 14476 Potsdam, Germany; 2 University of Amsterdam, Amsterdam, The Netherlands; 3 University of Heidelberg, Heidelberg, Germany

**Keywords:** Open data, Attentional control, SQL

## Abstract

Publicly available data are required to (1) assess the reproducibility of each individual findings in the literature, and (2) promote the reuse of data for a more efficient use of participants’ time and public resources. Current data-sharing efforts are well suited for the first goal, yet they do not sufficiently address the second goal. Here, we show how structured collections of open data can be useful, as they allow a larger community of researchers easy access to a large body of data from their own research area. We introduce the Attentional Control Data Collection, a SQL database for attentional control experiments. We illustrate the structure of the database, how it can be easily accessed using a Shiny app and an R-package, and how researchers can contribute data from their studies to the database. Finally, we conduct our own initial analysis of the 64 data sets in our database, assessing the reliability of individual differences. The analysis highlights that reliability is generally low, and provides insights into planning future studies. For example, researchers should consider increasing the number of trials per person and condition to at least 400. The analysis highlights how an open database like ACDC can aid meta-analytic efforts as well as methodological innovation.

Making data openly available has been a central demand by science reformers since the start of the reproducibility crisis in psychology (Nosek et al., 2015). Fortunately, this demand has led to a considerable increase in data availability. While only about 25% of data were shared after request in 2006 (Wicherts, Borsboom, Kats, & Molenaar, 2006), publicly sharing data upon publication is now more and more the norm. Between 2014 and 2021, the percentage of open data badges awarded at the journal *Psychological Science* drastically increased from 16 to 78% (Bauer, 2022). This cultural shift is also increasingly institutionalized: Universities and funding agencies prioritize open data, and some journals even mandate the publication of data with every published article (Sloman, 2015). In addition, technology like the Open Science Framework (OSF) and other data-sharing services enable an easy process for researchers, further reducing barriers to sharing data.

Data sharing serves two goals: (1) To make the scientific process more *transparent* and enable error and fraud detection, and (2) to make the scientific process more *efficient* by allowing data reuse for different research projects. Current data-sharing efforts, however, focus overwhelmingly on the first goal (Crüwell et al., 2023; Hardwicke et al., 2021; Obels, Lakens, Coles, Gottfried, & Green, 2020). We argue that, as a result, current data-sharing procedures do not sufficiently meet the second goal. Whenever researchers who are complying with common data-sharing procedures publish an article, they share the corresponding data on the OSF, ideally in a format that allows replicating the exact analyses reported in the article. The OSF repository is linked in the article, and readers may access the data through this link and check whether the analysis code and shared data correspond to the results section in the article. From an ethical standpoint, this process seems reasonable: Data shared through this process are often aggregated and cleaned, allowing the reproduction of results without sharing unnecessary details of participants’ behavior. However, one could also argue that this approach is unethical—failing to share raw, unaggregated data that is easily accessible and reusable constitutes a mismanagement of public resources and the time contributed by study participants. From this perspective, data need to be shared in a way such that researchers may not only reproduce the study’s findings but also investigate new research questions. This approach saves public resources because there is no need to unnecessarily collect similar data with each new research question. As a result, no additional participants need to be recruited who would waste their time reproducing data that already exists.

To enable data reuse, data sharing needs to be approached differently. Consider, for example, a researcher (like the first author of the current paper) might be interested in the Stroop task (Stroop, 1935). The Stroop task is popular in cognitive psychology (MacLeod, 1991), so we may assume that many studies include this or similar tasks in their studies. Instead of running yet another Stroop experiment, the researcher decides to use existing data to explore their research question before designing a more targeted study. First, the researcher needs to be able to find open Stroop task data. Currently, they could either search for papers on the topic and check whether open data are provided or search directly via OSF or other data-sharing servers. However, neither of these options is very promising, as the vast majority of articles in the literature still do not provide raw data, and data-sharing servers are not equipped with sufficient search options. Second, data sets need to be accessed easily and in a general, understandable format ready for reuse. There are data-sharing formats that provide this structure (Wilkinson et al., 2016), but they are not very common in psychological research. Moreover, data are usually shared on the level necessary for the original analysis. In the case of the Stroop task, researchers might share the Stroop effect per participant, but for this new analysis the researcher needs trial-level data. So again, there is yet another barrier to data reuse.

We think it is necessary to provide a data-sharing solution that solves the current problems and enables easy and efficient data reuse. Here, we propose to gather open data sets from a specific research area in a database based on Structured Query Language (SQL). This process requires little to no work from the authors of the original papers in addition to current data-sharing policies, some work from the lab(s) setting up the database, and little to no work from the researchers who wish to reuse open data. We describe the process and structure we used to set up a database of attentional control tasks called the Attentional Control Data Collection (ACDC). The database currently includes 64 data sets from 12 publications from tasks like the Stroop, Simon, and flanker tasks (see Appendix A). Subsequently, we show how researchers can contribute their own data to the database, how the data can be explored using a Shiny app, and accessed for reuse using an R-package. In an example analysis, we assess the reliability of the included tasks. This section highlights how an open database like ACDC can aid meta-analytic efforts as well as methodological innovation.

To provide a little history of the project, the Attentional Control Data Collection was inspired by a collection of open data sets by the Perception and Cognition Lab led by J. Rouder (url). Several researchers made data available to Haaf and Rouder to aid in the development of statistical modeling approaches (Haaf & Rouder, 2017; Rouder, Kumar, & Haaf, 2023). To ensure that data sets were accessible, Haaf and Rouder gathered them in a GitHub repository. However, there was little structure to the collection, and GitHub repositories are neither stable entities nor are they designed as data storage. Here, we describe how structured data collection can be achieved and which benefits it provides.

**Fig. 1:**
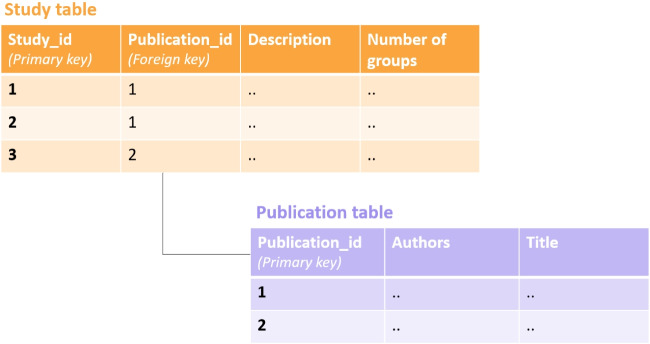
Illustrative example of using foreign and primary keys in a SQL database

Who is this good for? In general, the approach of collecting open data in databases can be useful for many researchers whose field regularly produces high-quality data that is difficult to find or access. The approach can be particularly useful in research areas where data acquisition is costly, either because of hard-to-reach populations or time-consuming data collection routines. Users of these databases may include researchers planning future studies who wish to evaluate common procedures, materials, and data patterns, methodological researchers who wish to apply their methods to empirical data, or researchers with meta-analytic questions. For our own collection, ACDC, we hope that it will serve the community of attentional control researchers to advance experimental and statistical methodology as well as theory development.

## SQLite database

One of the most standard ways in computer science for storing data is using an SQL database. SQL enables the creation, access, and manipulation of structured data storage. SQL databases are composed of data tables and the relationships between these tables. There are many flavors of SQL databases. Here, we decided to use an SQLite database, a lightweight solution that allows us to store the entire database in a single file of moderate size that can be downloaded by researchers for data reuse. In this section we describe the structure of the database and the data currently included. Researchers who simply want to use ACDC may safely skip this section.

### Database structure

SQL databases are composed of several data tables consisting of rows and columns. Each row in a data table has a primary key (essentially a row ID) which uniquely identifies it. To connect information in one table to information in another table in the database, the primary key in the first table can serve as a foreign key in the second table. This foreign key then references a specific row in the first table. In contrast to primary keys, foreign keys can have duplicate values, as long as they are unique in their primary table. For instance, a study table may store information about all studies in a database where each row corresponds to a single study. Here the primary key is the study_id. We can ensure that our database links each study to the publication it was published in by adding a foreign key called publication_id to the study table. This foreign key references the unique identifier of the respective publication in a publication table. Figure 1 illustrates the relationship between study table and publication table. While publication_id links to a single row in the publication table, it can occur several times in the study_table as there can be several studies per publication.

**Fig. 2:**
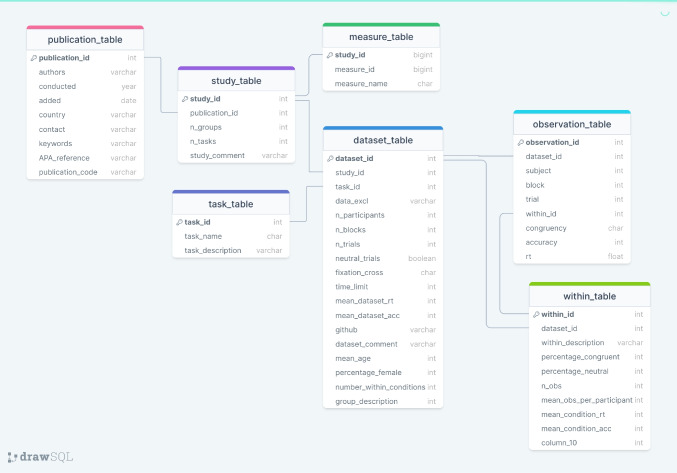
Structure of the ACDC database. Primary keys are indicated by the *key symbol*. References between data tables are illustrated through lines connecting columns across data tables. This overview includes the data type of each column: integers (int), numbers with decimal places (float), characters (varchar), and logical true/false values (booleans)

ACDC is adapted to the logic of publications consisting of one or multiple studies which in turn include one or more data sets. The whole structure of ACDC is shown in Fig. 2. A *publication table* and a *study table* contain specific information about each publication and study, respectively. Each data set within a study contains trial-level data from one attentional control task. If a between-subject manipulation exists, our database contains a separate data set for each group and each task. For instance, a study in which two groups (younger and older adults) completed a Simon and a Stroop task would consist of four data sets (i.e., younger-Stroop, younger-Simon, older-Stroop, older-Simon) in the ACDC database. An additional *measure table* tabulates which additional tasks and measures were used in the study that did not meet the criteria for inclusion of trial-level data in ACDC.

The *data set table* stores information about each data set, such as sample size, the number of within-participant manipulations, and whether a fixation cross was used. The *observation table* holds the trial-level data (including response time and accuracy). The task type of each data set (i.e., Stroop, Simon, Flanker, negative priming, or other) and a description of which stimuli were presented in the task are documented in the *task table*.

Within-participant manipulations are coded in the within ID column of the observation table. Further information about each condition of each data set (such as the percentage of congruent trials, mean response time, and mean accuracy) are recorded in the *within table*. Note that since the congruency of stimuli, i.e., whether response and stimulus attributes are compatible or incompatible, is part of every attentional control task, it is not considered a separate within-participant manipulation in this database but is per default included in the observation table.

### Data gathering

Data were gathered from a number of attentional control studies from the last several years that publicly shared their data. We did not conduct a systematic search for data sets nor attempted to distribute a wider call for open data. Instead, we included the data sets that were already made available to the lab for previous projects, and added data sets from collaborators bit by bit. This approach was chosen to make the project feasible, and to first set up a working database before large quantities of data are added.

The process for adding data was as follows: First, data repositories were screened for data files, analysis scripts and code book files to ensure that we understood each variable correctly. We then attempted to match key figures to the numbers reported in the corresponding papers. For example, we checked that sample sizes, number of trials per condition, and ranges of variables values matched the descriptions in the papers. If we found any discrepancies, we contacted the authors for clarification. We added the data set only if the discrepancies were resolved. We found discrepancies in most of studies in the database. Fortunately, all authors responded to our requests and we were able to resolve the majority of discrepancies. Nevertheless, for the limited number of data sets included, this process took several months.

We downloaded the raw data from open data repositories and then reshaped them into the required structure using R. Scripts from this process are available on GitHub: https://github.com/jstbcs/acdc-database/tree/main/Create_db/add_data/scripts_creating_list_objects. We integrated these transformed data with added meta-information about the publication into one list object per publication that mirrored the hierarchical data structure. We then used these lists to systematically add study information to all tables of the database (see https://github.com/jstbcs/acdc-database/blob/main/Create_db/reading_list_objects_to_db.R).

Until the date of submission of the manuscript, 64 data sets from 12 publications were included in the database. The full list of data sets and references is provided in the Appendix A. The current database includes data sets from studies with an experimental as well as a correlational focus. The data contain $10^ 7$ observations collected from 9978 participants.

### Contribute

We are planning to continuously add attentional control task data to our database. Researchers seeking to improve data accessibility and citation potential of their work are invited to submit their data and related study meta-information via our online form.

To be eligible for the ACDC database, the data must have been collected for a published or pre-registered study using an attentional control task where the amount of control needed is experimentally manipulated. Researchers submitting the data must be allowed to publicly share them in an anonymized format. Furthermore, the data files have to contain trial-level information on anonymized subject IDs, reaction time, accuracy (i.e., correct/ incorrect), and a congruency variable, indicating whether distractor stimuli were congruent, conflicting, or neutral. In the case of between-subject manipulations and within-subject manipulations (besides congruency), the files should contain a between and a within variable indicating which condition a trial belonged to.

When submitting data to our online submission form, re-searchers will be asked to provide meta-information about the publication and study, such as descriptions of the attentional control task and the between and within manipulations. Data files can either be uploaded in openly readable data formats or as a link to a repository, such as OSF or GitHub.

## Accessing the database

One advantage of SQLite databases is that they are simply a file that can be downloaded and locally accessed by anyone. Our database is provided in a GitHub repository.^1^ To access the database, researchers can download the file acdc.db, and use the SQLite tool of their choice. In addition, we built R-based tools to inspect, access, and select data from ACDC. We introduce these tools, a Shiny app and an R package, subsequently.

### Shiny app

**Fig. 3:**
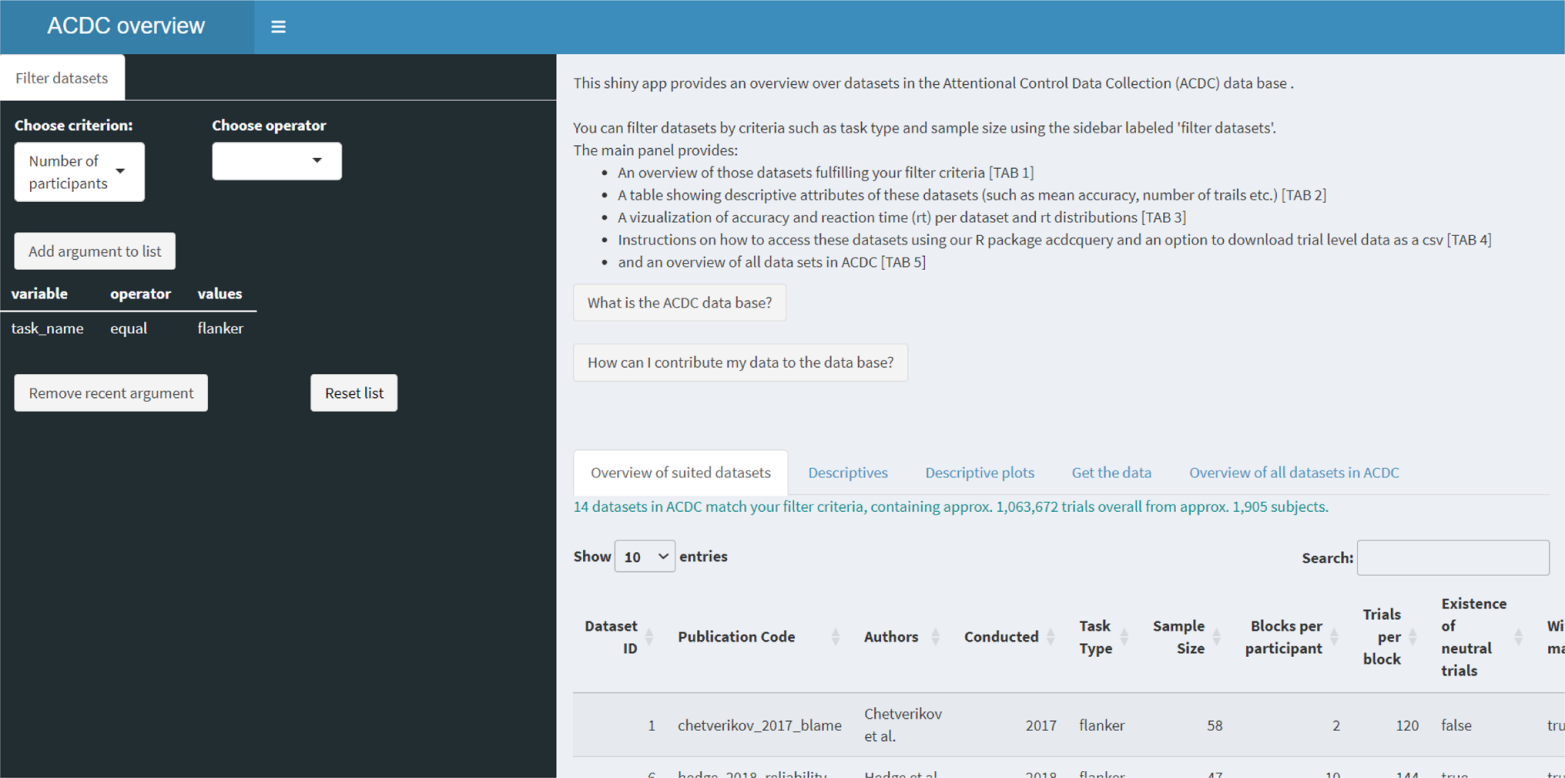
Interface of the ACDC Shiny app. The side bar allows users to choose a criterion, operator, and value to filter data sets. Chosen arguments are listed in the sidebar below and can be removed using the respective fields. The main panel shows general information about the app and the ACDC project, and provides detailed information about the filtered data sets and how to access the data

The easiest way to inspect the data is using our Shiny app provided here. The interface of the app is shown in Fig. 3. The app allows inspecting all data sets or to select data sets with certain specifications using the filter box on the left. For example, if a researcher is interested in the flanker task, they may select all flanker data sets for closer inspection. After selection, researchers can choose between an overview of the included data sets, some descriptive statistics, and descriptive plots. If they want to further analyze the data, they can download the data via the “get the data” tab, either directly as csv file or using the provided R-code.

Note that all descriptive statistics in the Shiny app are aggregated across congruency conditions. This was a deliberate choice when designing the app. By withholding information about the effect of interest, researchers can inspect and select the data based on varying characteristics (including distributional properties), but remain unbiased as to the most relevant outcome variable. We hope that researchers can then formulate (and perhaps preregister) hypotheses about their reanalysis without much hindsight bias.

### R-Package

The R-package acdcquery allows a more customized but still user-friendly interaction with the database without ever writing any SQL code (Lesche, Hoffstadt, & Haaf, 2023).^2^ Using three key functions, connect_to_db(), add_argument(), and query_db(), the package en-ables connecting to the database, filtering data based on any variable, and loading specified variables as a data frame into R. The add_argument() function allows easy specification of filter arguments. Any variable present in any table in the database may be used as a filter condition in the query. Users may also provide multiple arguments and control which of them should be combined using *AND* and which should be combined using *OR* logic. Much as any variable in the database can be used as a filter, any variable or combination of variables can also serve as an output of the filtering process. The package constructs one SQL query combining all query arguments and the requested output variables. If variables from multiple tables are requested, or if the query selection is based on variables present in multiple tables, the SQL query will first join all involved tables together in a smart way. Specifically, the query is optimized for speed by automatically discarding unnecessary variables, choosing efficient ways to join tables, and eliminating the need for temporary storage of query results. All arguments and requests are combined simultaneously, allowing optimal use of SQLite’s built-in optimization.

### Queries and output

Below, we will illustrate several examples for using the R-package. Querying the database consists of five steps:

1. Connecting to the database,
2. specifying the filter arguments,
3. specifying the relationship of filter arguments,
4. specifying the output variables,
5. and querying the database.

For the first step, the package is installed from CRAN and loaded. Note that the database also needs to be downloaded for this step. A connection to the database is established using connect_to_db().
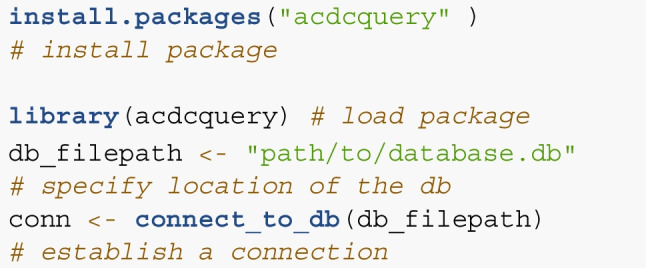


For the second step, the function add_argument() is used to specify a list containing query arguments. In the following code chunk, we specify three arguments. Typically, function either builds the SQL query from the provided input, however, it is also possible to manually submit an SQL statement (see the last argument).
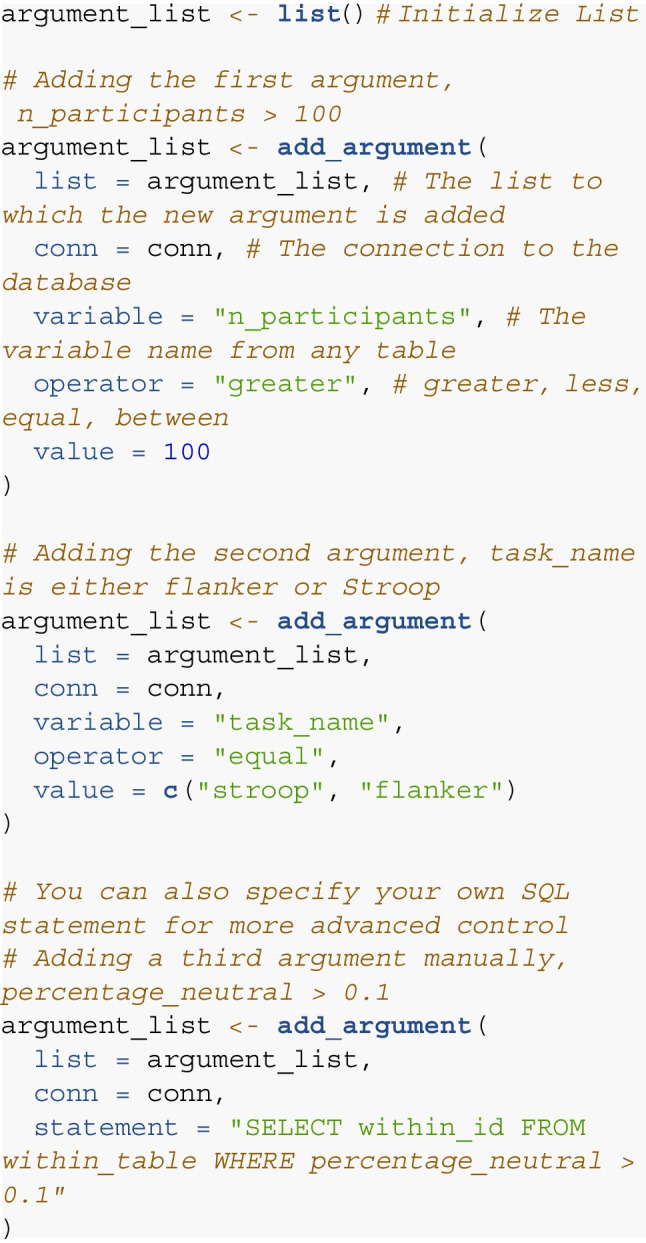


This argument list now contains three separate filter arguments. In step 3, we specify which arguments should be combined using *AND* or using *OR*. In our case, we only want those cases for which all are true, which corresponds to the specification all_true.
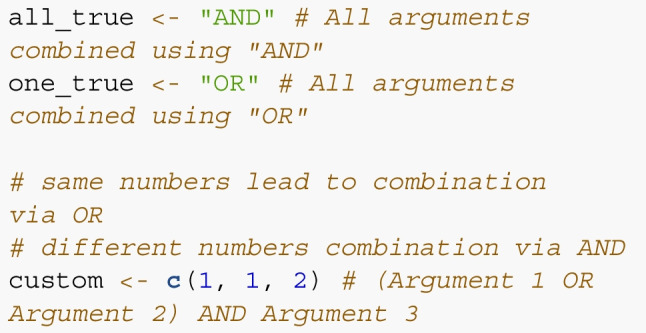


In step 4, we specify which variables should be returned by the query. Here, any variable present in any table can be selected. To allow for fast queries in the case of limited involvement of multiple tables, the user can specify the table the query is centered on using the target_table argument of query_db(). In most cases, this is the observation table containing trial data. If dataset-level data is of interest, it is wise to specify target_table = dataset_table. If all variables of the target table and some additional variables from other tables should be returned, simply add “default” to the vector specifying the target variables.
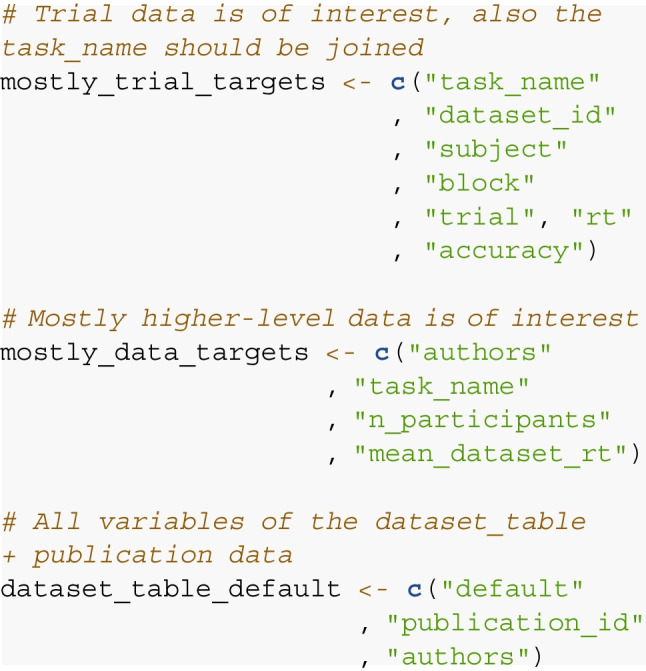


Finally, in step 5’s argument list, the relationship of arguments, target variables, and target table are supplied to the query_db() function.
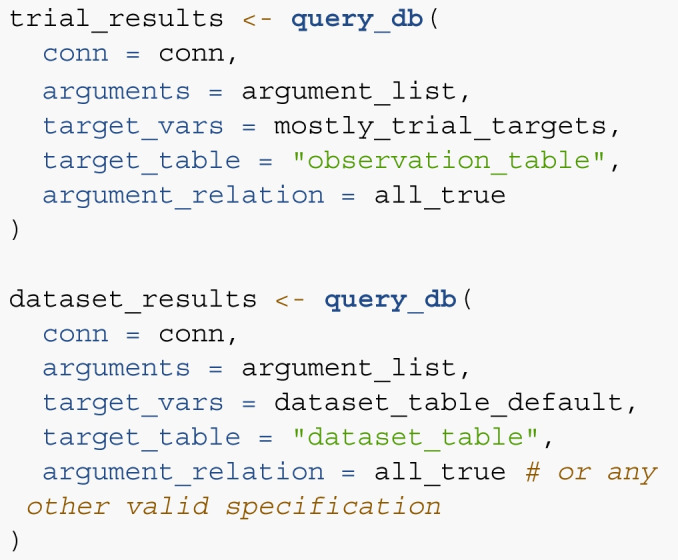


Additional info can be found on the package’s GitHub page.

## Reliability of ACDC tasks

To illustrate how ACDC can be used for reanalysis, we provide an example data analysis assessing the reliability of attentional control tasks.^3^ Reliability of tasks like the Stroop task has recently been the target of much debate (Draheim, Mashburn, Martin, & Engle, 2019; e.g., Hedge, Powell, & Sumner, 2018; Rey-Mermet, Gade, & Oberauer, 2018; Rouder & Haaf, 2019; Rouder et al., 2023). This debate spans both the observation that the reliability of individual differences in these tasks is generally low (e.g., Hedge et al., 2018; Rouder et al., 2023), and whether the methods for estimating reliability are appropriate (e.g., Rey-Mermet, Singmann, & Oberauer, n.d.; Rouder & Haaf, 2019). Rouder et al. (2023) aimed to assess the reliability of individual differences in attentional control tasks more globally and with improved statistical methods. To this end, they conducted an analysis of 24 data sets (see their Table 2). The current analysis is an extension of the analysis by Rouder et al. (2023) with more than twice the number of tasks.

Here, we rely on methodological development by Rouder and colleagues (Rouder & Haaf, 2019; Rouder et al., 2023; Rouder & Mehrvarz, 2024) to survey the reliability of data sets in ACDC. The authors suggested improving the analysis of reliability of cognitive tasks based on two simple observations. First, conventional measures of reliability such as the split-half reliability or intra-class correlation are not well suited for cognitive tasks as they are dependent on the number of trials. The reason for this dependency is that observed between-subjects variability of a target effect is a combination of true individual differences and sample noise. If sample noise is large, as is the case in these tasks, reliability can be drastically reduced with a small number of trials compared to a large number of trials. Second, unlike with psychometric tests, the number of trials of cognitive tasks is oftentimes chosen arbitrarily, as a matter of preference by the lab conducting the experiment. These two issues, taken together, make it difficult to compare reliability across labs or across tasks (Rouder & Haaf, 2019).

**Fig. 4:**
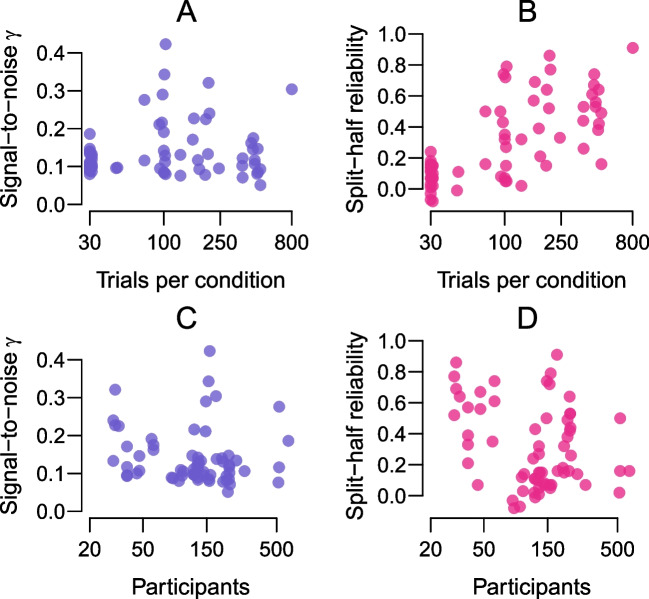
Reliability estimates as a function of number of trials and participants. **A.** The signal-to-noise ratio is independent of the (log) number of trials per condition. **B.** The split-half reliability is highly correlated with the (log) number of trials per condition. **C.** The signal-to-noise ratio is independent of the (log) number of participants. **D.** The split-half reliability has a slight negative relationship with (log) number of participants due to the fact that large participants studies commonly use a smaller number of trials per task

The relationship between common reliability estimates and trial number is illustrated in Fig. 4B. The figure shows the split-half reliability coefficients for all ACDC data sets as a function of the (log) number of trials per condition. The correlation between split-half-reliability and log trial number is $r = 0.65$, 95% HDI $[0.50, 0.78]$, $\textrm{BF}_{\text {10}} = 2.62 \times 10^{7}$. Figure 4D shows the relationship between split-half reliability coefficients and the (log) number of participants. Theoretically, there should be no relationship. However, for purely practical reasons, many studies with a large number of participants have in turn a low number of trials. For example, one of the studies with the largest number of participants, the one by Enkavi et al. (2019), only has 73 trials per condition for the Stroop and Simon task. Therefore, empirically, we do find a negative relationship between the log number of participants and split-half reliability, $r = -0.26$, 95% HDI $[-0.47, -0.03]$, $\textrm{BF}_{\text {10}} = 3.41$.

To counter the lack of comparability of conventional reliability measures, Rouder et al. (2023) proposed a measure of reliability that is derived from hierarchical modeling of the trial-level data. This modeling approach allows to separately estimate true individual variability and sample noise. As a result, we may determine how much true variability there is relative to variability due to sample noise. This ratio, termed $\gamma ^2$, can be expressed as

$$\begin{aligned} \gamma ^2 = \frac{\sigma ^2_{\text {true}}}{\sigma ^2_{\text {noise}}}, \end{aligned}$$

where the numerator is the true variance of individuals’ effects estimated by the model, and the denominator is the trial-by-trial within-person variability. For the purpose of individual differences research, $\sigma ^2_{\text {true}}$ represents the signal and $\sigma ^2_{\text {noise}}$ represents noise, therefore, Rouder and colleagues termed $\gamma ^2$ the signal-to-noise ratio. Here, we use the square root of $\gamma ^2$, $\gamma$, representing a ratio of standard deviations as researchers tend to be more familiar with standard deviations rather than variances.

**Fig. 5:**
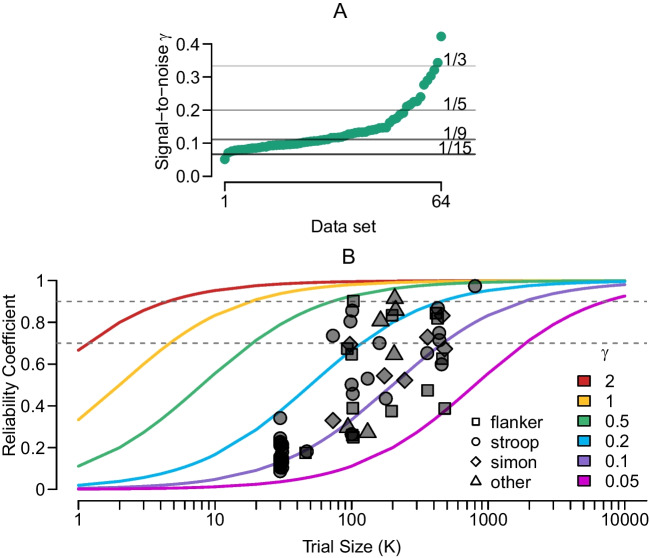
Signal-to-noise ratio and reliability. **A.** Signal-to-noise ratio $\gamma$ for all data sets ordered from smallest to largest. **B.** Reliability as a function of number of trials for all data sets in ACDC. The *lines* represent different signal-to-noise ratios. For a fixed $\gamma$, the relationship between the number of trials per condition and the reliability is deterministic

Figure 5A shows the signal-to-noise ratio $\gamma$ for all data sets ordered from smallest to largest. The median of $\gamma$ is $Med = 0.12$ corresponding to a ratio of roughly 1 to 8. That is, for every unit of signal there are 8 units of noise. Using the ACDC data sets, we can also assess the independence of $\gamma$ from the number of trials and participants. Figure 4A and C show these relationships. There is no correlation between the log number of participants and $\gamma$, $r = -0.12$, 95% HDI $[-0.32, 0.13]$, $\textrm{BF}_{\text {10}} = 0.44$, nor between the log number of trials and $\gamma$, $r = 0.12$, 95% HDI $[-0.09, 0.36]$, $\textrm{BF}_{\text {10}} = 0.49$.

Overall, the assessment of reliability of the currently used attentional control tasks shows mixed results. Signal-to-noise ratios of these tasks tend to be around 0.1–0.2, highlighting overwhelming levels of trial-by-trial variability compared to true individual variability of congruency effects. As shown in Fig. 5B, there is no task type standing out with consistently higher or lower reliability. That is, there is no argument based on reliability that would make us prefer a specific task, for example, a flanker task over a Stroop task. However, the analysis can still help us plan future studies.

Figure 5B shows the functional relationship between the reliability coefficient and the number of trials through the signal-to-noise ratio. We can follow the lines to understand which number of trials would yield an acceptable reliability for a task with a certain signal-to-noise ratio. For tasks with a signal-to-noise ratio around 0.2, around 100 trials per condition and person are needed for acceptable levels of reliability. For tasks with a signal-to-noise ratio around 0.1, roughly 500 trials are needed.

We also see that all studies with large numbers of trials have acceptable levels of reliability. These insights match a recent finding by Lee et al. (2023). The authors had a small number of participants go through almost 4000 trials per condition per task to assess how many trials are needed for precise estimates of individual effects. They conclude that around 400 trials per person and condition (or 800 per person across two conditions) are needed for stable estimates of individual effects. From our current analysis using a larger number of studies and participants, we can confirm the results by Lee et al. (2023) and Rouder et al. (2023).

## Conclusion

Open data policies are required to (1) to assess how many results in the literature are reproducible, and (2) to promote the reuse of data for a more efficient use of participants’ time and public resources. While repositories like the Open Science Framework are well suited for the former goal, they do, at this point, not sufficiently address the latter goal. We argue that structured collections of open data are much more supportive of this goal as they allow a larger community of researchers easy access to a large body of data. Here, we introduce the Attentional Control Data Collection, an SQL database for attentional control experiments. The database is easy to access using a Shiny app and an R-package, and is built to grow whenever researchers are willing to contribute their data in the future. We illustrate the structure of the database, how it can be accessed, and provide an example for statistical analyses that can be conducted with such a large data collection.

The database approach is related to meta-analysis, but different in two crucial aspects. First, it does not represent a systematic overview, or even a representative sample of studies. To remedy this potential issue, researchers planning to develop an open data collection could conduct a systematic review of the literature including *all* studies with open data that meet their criteria in the database. However, as the field only recently started sharing data, older studies would still be systematically excluded, and there might still remain systematic differences between more recent studies that do and do not share data. Second, the database approach enables much more sophisticated research questions and analyses. As the database provides raw, unfiltered, trial-level data, we may use it for studying questions far beyond congruency effects.

Here, we illustrated this potential by fitting Bayesian hierarchical models to all data sets in the database. With this analysis, we were able to study the reliability of individual differences, something that is rarely done in the field of cognitive psychology. Yet, there are many more approaches that could be applied, tested, or refined by applying them to a larger body of data. Potential analyses target sequential effects, computational modeling, or even methodological questions like the effect of online versus in-lab data collection on data quality. Another potential application for the database is to extract information about expected values and variability of parameters in attentional control tasks. For example, van Erp, Verhagen, Grasman, and Wagenmakers (2017) developed informed prior distributions for meta-analysis by surveying 705 published meta-analyses. Similarly, one could develop prior distributions for response time models using the data in our database. These expected parameter values may also be used for simulation studies with realistic values.

In addition to providing a tool for attentional control researchers, ACDC may serve as a blueprint for researchers from other areas to build their own data collections. The database structure, R package and Shiny app can be easily adapted and maintained by a single lab with an invested interest in open data. The scope of these data collections may vary by field and capacity of the researchers building these collections. For our purposes, we designed ACDC to include data sets with very similar experimental designs and focus. Yet, it is plausible that the scope may be widened depending on the area or research questions that ought to be investigated using the data collections. Technically, such a data collection could have a wider focus, such as all data collected by a department or university, all data in a certain field, or even all data on the Open Science Framework. However, such collections would require a lot of maintenance and technical support, and they would not be manageable with our setup. SQLite files become quite large very quickly, so that it might be useful to move to a proper SQL database when data collections grow. Additionally, if the data sets become too dissimilar, it might be helpful to switch to a different database structure such as non-relational databases. These databases provide less structure, which makes it more difficult to access relevant information, but scale better for more loosely organized data collections.

Finally, we hope researchers are willing to contribute data from their studies to ACDC or other open data collections. If they already share their data on the OSF, we see no disadvantage in also contributing to more structured data collections. In the best case, their data will contribute to advancing the field without requiring unnecessary additional data collection, leading to faster insights based on more data, and as for a personal benefit, sharing raw data in a larger collection might even boost the citation record of their study.

## Open Practices Statement

- All data are available in the ACDC database: https://github.com/jstbcs/acdc-database/blob/main/acdc.db.
- All code for creating the database is available on GitHub: https://github.com/jstbcs/acdc-database/tree/main/Create_db.
- The R-package is available on GitHub: https://github.com/SLesche/acdc-query.
- The manuscript and analysis code for the analysis illustration are available on GitHub: https://github.com/jstbcs/acdc-paper.

## Data Availability

All data are available in the ACDC database.

## Appendix A: All ACDC data sets

Table 1 contains all data sets that are part of the Attentional Control Data Collection at the time of manuscript submission. This version of the database is marked by a git tag, and the git repository for the tag is linked here. The table is ordered by dataset ID and study, and contains the number of trials per condition and participant, and the number of participants. Additionally, three reliability estimates are included: The signal-to-noise ratio, $\gamma$, the split-half reliability for 2000 random splits, $\rho$, and the corrected split-half reliability using the Spearman–Brown formula, $\rho ^*$.

**Table 1:** All datasets currently in ACDC

ID	Task name	I	K	$\gamma$	$\rho$	$\rho ^*$
Chetverikov et al. (2017)						
1	Flanker	58	100	0.191	0.35	0.51
Enkavi et al. (2019)						
2	Stroop	523	73	0.276	0.50	0.67
3	Simon	522	73	0.116	0.16	0.28
4	Other	516	131	0.076	0.02	0.04
Hedge et al. (2018)						
5	Stroop	47	439	0.107	0.56	0.72
6	Flanker	47	426	0.146	0.67	0.80
7	Stroop	60	428	0.175	0.74	0.85
8	Flanker	60	414	0.162	0.61	0.75
Kucina et al. (2023)						
9	Other	31	163	0.227	0.69	0.82
10	Other	30	205	0.133	0.52	0.68
11	Other	31	207	0.321	0.86	0.92
12	Flanker	33	198	0.225	0.64	0.77
13	Other	30	210	0.240	0.77	0.87
Löffler, Frischkorn, Hagemann, Sadus, and Schubert (2022)						
14	Stroop	148	98	0.290	0.74	0.85
15	Flanker	147	93	0.211	0.50	0.67
16	Negative priming	142	94	0.095	0.08	0.13
Ebersole et al. (2016)						
17	Stroop	160	30	0.096	0.07	0.13
18	Stroop	288	31	0.106	0.07	0.12
19	Stroop	136	31	0.133	0.15	0.26
20	Stroop	195	30	0.140	0.18	0.30
21	Stroop	93	30	0.080	–0.07	–0.13
22	Stroop	117	30	0.147	0.24	0.38
23	Stroop	127	31	0.119	0.10	0.18
24	Stroop	142	30	0.104	0.07	0.12
25	Stroop	131	31	0.127	0.15	0.25
26	Stroop	82	30	0.089	–0.03	–0.05
27	Stroop	98	30	0.094	0.03	0.05
28	Stroop	119	31	0.098	0.02	0.03
29	Stroop	100	30	0.105	0.14	0.24
30	Stroop	120	30	0.111	0.11	0.18
31	Stroop	613	30	0.186	0.16	0.28
32	Stroop	96	30	0.109	0.12	0.20
33	Stroop	178	30	0.138	0.16	0.27
34	Stroop	84	31	0.087	–0.08	–0.13
35	Stroop	45	31	0.100	0.07	0.12
36	Stroop	250	30	0.134	0.14	0.25
37	Stroop	129	30	0.089	0.01	0.03
Pratte, Rouder, Morey, and Feng (2010)						
38	Simon	38	245	0.095	0.33	0.48
39	Stroop	38	160	0.171	0.57	0.72
40	Simon	38	174	0.117	0.39	0.55
41	Stroop	38	178	0.093	0.21	0.33
Rey-Mermet et al. (2018)						
42	Stroop	129	102	0.129	0.27	0.42
43	Stroop	129	100	0.142	0.32	0.48
44	Flanker	129	102	0.112	0.15	0.25
45	Flanker	129	101	0.083	0.05	0.09
46	Stroop	155	101	0.343	0.72	0.83
47	Stroop	155	100	0.085	0.07	0.13
48	Flanker	158	103	0.423	0.79	0.88
49	Flanker	157	103	0.080	0.05	0.10
Stahl et al. (2014)						
50	Stroop	199	131	0.131	0.32	0.48
51	Flanker	199	196	0.078	0.15	0.26
Tang, Bugg, Snijder, Conway, and Braver (2023)						
52	Stroop	176	801	0.304	0.91	0.95
Von Bastian, Souza, and Gade (2016)						
53	Stroop	121	47	0.097	0.11	0.18
54	Simon	121	97	0.216	0.43	0.60
55	Flanker	121	46	0.096	–0.01	–0.02
Whitehead, Brewer, and Blais (2019)						
56	Stroop	221	438	0.117	0.53	0.69
57	Simon	219	460	0.147	0.64	0.78
58	Flanker	220	462	0.085	0.42	0.58
59	Stroop	211	456	0.081	0.38	0.55
60	Simon	211	480	0.093	0.49	0.65
61	Flanker	216	481	0.051	0.16	0.26
62	Stroop	222	358	0.102	0.44	0.61
63	Simon	221	360	0.122	0.53	0.69
64	Flanker	224	360	0.071	0.26	0.41

*Note.* $\gamma =$ signal-to-noise ratio; $I =$ number of participants; $K =$ number of trials per condition; $\rho =$ split-half reliability coefficient; $\rho ^* =$ Spearman-Brown corrected reliability coefficient

## Appendix B: R-packages for analysis

All analyses were conducted using the following R packages: R (Version 4.4.2; R Core Team, 2022) and the R-packages *acdcquery* (Version 1.0.1; Lesche et al., 2023), *BayesFactor* (Version 0.9.12.4.7; Morey & Rouder, 2023), *coda* (Version 0.19.4.1; Plummer, Best, Cowles, & Vines, 2006), *DBI* (Version 1.2.3; R Special Interest Group on Databases (R-SIG-DB), Wickham, & Müller, 2022), *dplyr* (Version 1.1.4; Wickham, François, Henry, Müller, & Vaughan, 2023), *DT* (Version 0.33; Xie, Cheng, & Tan, 2023), *forcats* (Version 1.0.0; Wickham, 2023), *ggplot2* (Version 3.5.1; Wickham, 2016), *htmltools* (Version 0.5.8.1; Cheng et al., 2023), *knitr* (Version 1.49; Xie, 2015), *lubridate* (Version 1.9.4; Grolemund & Wickham, 2011), *MASS* (Version 7.3.61; Venables & Ripley, 2002), *Matrix* (Version 1.7.1; Bates, Maechler, & Jagan, 2023), *MCMCpack* (Version 1.7.0; Martin, Quinn, & Park, 2011), *papaja* (Version 0.1.3; Aust & Barth, 2022), *purrr* (Version 1.0.2; Wickham & Henry, 2023), *readr* (Version 2.1.5; Wickham, Hester, & Bryan, 2023), *RSQLite* (Version 2.3.9; Müller, Wickham, James, & Falcon, 2023), *shiny* (Version 1.10.0; Chang et al., 2023), *splithalf* (Version 0.8.2; Parsons, 2021), *stringr* (Version 1.5.1; Wickham, 2022), *tibble* (Version 3.2.1; Müller & Wickham, 2023), *tidyr* (Version 1.3.1; Wickham, Vaughan, & Girlich, 2023), *tidyverse* (Version 2.0.0; Wickham et al., 2019) and *tinylabels* (Version 0.2.5; Barth, 2022).

## Footnotes

1 The newest version can be accessed via https://github.com/jstbcs/acdc-database/blob/main/acdc.db, the version at the time of submission can be found here.

2 Note that the R package is not developed to build a new SQL database but solely to interact with ACDC.

3 For a full list of R-packages used for the analyses see Appendix B.

## Code Availability

All code is available on GitHub

## Declarations

### Code Availability

All code is available on GitHub.
